# Comparative Evaluation of En Bloc Staining Protocols for Deep-Learning Segmentation of Dorsal Root Ganglia in SBF-SEM

**DOI:** 10.3390/jimaging12100473

**Published:** 2026-09-30

**Authors:** Vitalijs Borisovs, Mario Bossi, Laura Matino, Paola Marmiroli, Guido Cavaletti

**Affiliations:** 1 Experimental Neurology Unit, School of Medicine and Surgery, Università di Milano-Bicocca, 20900 Monza, Italy; 2 Istituto Degli Endotipi in Oncologia, Metabolismo e Immunologia “Gaetano Salvatore” (IEOMI), Consiglio Nazionale Delle Ricerche, 80131 Naples, Italy; 3 Fondazione IRCCS San Gerardo Dei Tintori, 20900 Monza, Italy

**Keywords:** volume electron microscopy, deep learning segmentation, dorsal root ganglia, en bloc staining, image preprocessing

## Abstract

The integration of deep learning algorithms into volumetric electron microscopy can be limited by biochemical sample preparation. En bloc staining protocols, emphasizing heavy-metal contrast, are optimized for the convenience of human visual interpretation. This study evaluates the suitability of three staining protocols for automated segmentation and 3D reconstruction of dorsal root ganglia (DRG): rOTO-based heavy-metal staining (Ellisman), standard transmission electron microscopy (TEM) staining (Palade), and uranyl-free en bloc staining (X-solution). U-Net-based deep learning models were applied to dorsal root ganglia (DRG) samples to segment nuclei, mitochondria, satellite glial cells (SGCs), and the endoplasmic reticulum (ER). Quantitative segmentation performance was evaluated using the Dice similarity coefficient (DSC) and intersection over union (IoU) for each segmented structure group based on the comparison between the automated prediction masks and 30 manually annotated ground truth (GT) sections per staining protocol. Although the Ellisman-stained samples produced strong ultrastructural contrast, more false-positive and false-negative regions were observed for several structures. The Palade protocol provided insufficient membrane contrast to reliably identify the ER in this dataset. In contrast, the uranyl-free protocol produced the highest DSC and IoU values for SGCs and mitochondria and higher ER segmentation scores than the Ellisman protocol.

## 1. Introduction

Understanding the three-dimensional (3D) cellular architecture of the peripheral nervous system is important for characterizing the morphological basis of sensory function and neuropathology [1,2]. The dorsal root ganglion (DRG), a complex heterogeneous tissue comprising pseudo-unipolar sensory neurons encapsulated by satellite glial cells (SGCs), is challenging to image using volumetric electron microscopy (vEM) because of its high lipid content and complex membrane interdigitations [1,3]. Although SGCs were historically regarded primarily as metabolic support cells, recent ultrastructural studies suggest that the DRG is also involved in complex intraganglionic communication, mediated largely by the interaction between the sensory neuron soma and its enveloping SGCs [2,4,5]. The spatial relationship between the neuron and surrounding SGCs is complex, forming a tight, multilayered sheath around the neuron and extending numerous microvilli that interdigitate with the neuronal plasma membrane [1,3]. Characterizing these spatial relationships requires imaging approaches capable of reconstructing larger tissue volumes than can be readily obtained from conventional TEM sections [6]. While TEM has provided insights into organelle ultrastructure since the mid-20th century, its reliance on ultrathin 2D sections makes analysis of long-range axonal continuity and large-scale organelle organization more laborious and technically demanding [6].

To address the limitations of 2D imaging, volumetric electron microscopy (vEM) has become an important approach in neurobiology for reconstructing cellular structures in three dimensions [7,8]. Techniques such as serial block-face scanning electron microscopy (SBF-SEM) and focused ion beam scanning electron microscopy (FIB-SEM) allow for the automated acquisition of aligned image stacks with isotropic or near-isotropic voxel resolution [8,9,10]. In SBF-SEM, a diamond knife mounted within the SEM vacuum chamber physically removes the surface of a resin-embedded block face after imaging, exposing a fresh surface for the subsequent scan. This process, repeated thousands of times, generates datasets capable of resolving the 3D connectivity of neural circuits [11,12]. The image contrast generated in SBF-SEM also differs from that obtained in conventional TEM. SBF-SEM relies on the detection of backscattered electrons (BSEs) generated from the block surface, the yield of which is directly proportional to the mean atomic number (Z) of the atoms within the interaction volume. Consequently, the contrast in SBF-SEM images is strongly influenced by the distribution and concentration of heavy metals (osmium, uranium, lead) bound to biological structures [13]. Furthermore, because the electron beam injects charge into the insulating epoxy resin, the sample must possess sufficient electrical conductivity to dissipate this charge to ground. Insufficient conductivity results in “charging artifacts,” where the accumulation of electrons deflects the primary beam, which can substantially reduce the accuracy and reliability of automated segmentation [6,9,10,12].

The requirement for high-atomic-number contrast and electrical conductivity has necessitated the development of aggressive “en bloc” staining protocols. In the 1950s, George Palade and colleagues established osmium-based fixation and staining approaches that enabled detailed visualization of cellular membranes and organelles by TEM [14,15]. The “Palade protocol” became the standard for TEM, providing exquisite resolution of lipid bilayers and organelles; however, for SBF-SEM, it is often insufficient. It lacks the deep heavy metal penetration required for significant backscatter yield and fails to render the sample conductive enough to prevent charging under the high-energy electron beams used in serial imaging [16,17,18]. To overcome the conductivity bottleneck, the National Center for Microscopy and Imaging Research (NCMIR) developed a combinatorial heavy-metal staining protocol [18]. This method utilizes a “ferrocyanide-reduced osmium-thiocarbohydrazide-osmium” (rOTO) bridging technique, combined with en bloc uranyl acetate and lead aspartate staining at elevated temperatures, to increase heavy-metal deposition throughout the tissue and improve both backscattered-electron contrast and conductivity [5,17,18]. The Ellisman protocol is widely used for SBF-SEM. However, it involves hazardous reagents, is labor-intensive (typically requiring 4–5 days), and can be prone to precipitation artifacts if the staining steps are not carefully controlled [12,13,16].

Alternative staining strategies have also been developed for large-volume neural tissue imaging. The BROPA (brain-wide reduced-osmium staining with pyrogallol-mediated amplification) protocol was introduced to achieve homogeneous heavy-metal staining of large tissue volumes for electron microscopy-based connectomics [19,20]. A subsequent modification, fast-BROPA (fBROPA), reduced processing time and provided a uranyl-acetate-free approach for volume electron microscopy [19,20]. These methods represent an important parallel development in the optimization of en bloc staining for large-scale volumetric imaging.

Concerns regarding the handling and regulation of radioactive materials have further motivated the development of non-radioactive alternatives utilizing lanthanides (e.g., ytterbium, samarium, gadolinium) [21,22,23]. The “X-solution,” a commercial formulation based on ytterbium (Yb3+) and phosphotungstic acid (PTA), has shown promise as a high-contrast, non-toxic alternative [24]. However, its suitability for automated segmentation of complex tissues such as the DRG has not been established. This study compares three staining methods applied to the 3D reconstruction of dorsal root ganglia. We specifically address segmentation fidelity, operational efficiency, and technical feasibility. We also present a 3D reconstruction of a DRG sensory neuron unit.

## 2. Materials and Methods

### 2.1. Biological Specimens and Staining Protocols

Three archived dorsal root ganglion (DRG) SBF-SEM datasets were analyzed in the present study, corresponding to three different en bloc staining preparations. The specimens originated from healthy BALB/c control mice and were generated as part of the experimental series previously described in detail [25]. Briefly, the three datasets corresponded to: (1) a modified Palade protocol based on buffered osmium tetroxide; (2) an Ellisman rOTO-based heavy-metal protocol utilizing osmium-thiocarbohydrazide-osmium bridging; and (3) a uranyl-free preparation utilizing the lanthanide-based X-solution.

The datasets used here were retrospectively selected from the archived DRG material generated for the previous study [25]. Because the specimens had been processed as part of a broader experimental series before the present analysis, and complete historical sample-tracking information is no longer available, the exact numbers of source animals, DRGs, resin blocks, and independent staining sessions represented in the original specimen collection could not be reliably reconstructed. The present comparison should therefore be interpreted as a technical comparison of three volumetric datasets, one for each staining preparation, rather than as a biologically replicated comparison of staining protocols.

Primary serial block-face scanning electron microscopy (SBF-SEM) image acquisition was performed using a Zeiss GeminiSEM 360 (Carl Zeiss S.p.A., Milan, Italy) equipped with an in-chamber ultramicrotome (Volutome) and a backscattered electron (BSE) detector. Acquisitions were standardized to a slice thickness (Z-step) of 50 nm. The native voxel dimensions were 7 × 7 × 50 nm^3^ for the Palade dataset, 5 × 5 × 50 nm^3^ for the Ellisman dataset, and 6 × 6 × 50 nm^3^ for the uranyl-free X-solution dataset. The resulting full image stacks comprised 400 images for the Palade dataset, 201 images for the Ellisman dataset, and 221 images for the uranyl-free dataset. To conserve computational resources during subsequent processing and rendering, the raw volumetric stacks were cropped to isolate regions of interest (ROIs) containing at least one complete, fully encapsulated DRG sensory neuron. Scale bars were generated using the calibrated native voxel dimensions of each dataset and therefore represent physical distances independently of differences in lateral pixel size.

### 2.2. Computational Workflow and Pre-Processing Script

The core of the computational workflow relies on a custom Python (v3.14.5) script integrated via the Python Application Programming Interface (API) within the ZEISS Arivis Pro environment. All raw image stacks were ingested into Arivis Pro, where the script executed a multi-stage preprocessing sequence to standardize the disparate data domains before neural network inference.

To manage variant dynamic ranges and mitigate charging artifacts, the script first applies non-local means denoising, which effectively suppresses stochastic noise while preserving the high-frequency edge information critical for detecting delicate structures like the satellite glial cell (SGC) sheath and the endoplasmic reticulum (ER). Following denoising, the script performs global Z-score normalization across the volumetric stack, calculated as

(1) $$\bar{x}=\frac{x\;-\;\mu }{\sigma +\varepsilon }$$

where *μ* and *σ* represent the global mean and standard deviation of the volume, and *ε* is a stability constant (1 × 10^−5^).

All datasets were processed using the same fixed preprocessing workflow before model inference. The workflow comprised non-local means denoising, global Z-score normalization, CLAHE (clip limit 3.0; tile grid size 8 × 8), and sharpening. These operations were applied to reduce noise, normalize global intensity ranges, enhance local contrast, and accentuate membrane boundaries. The individual contribution of each preprocessing operation was not evaluated in the present DRG study. Accordingly, the protocol comparison represents performance under the common preprocessing and segmentation workflow and should not be interpreted as an isolated effect of staining chemistry independent of preprocessing. The rationale and previous evaluation of this preprocessing strategy have been described previously [26].

### 2.3. Model Training and Evaluation Metrics

Model training employed an iterative, active-learning strategy natively supported by the ZEISS Arivis Pro environment. A foundational training set of highly diverse, representative image planes from all three sample preparations (Palade, Uranyl-free, and Ellisman) was curated. Ground truth (GT) annotations were manually generated across this composite set to capture the full spectrum of morphological variability for four specific DRG domains: satellite glial cells (SGCs), neuronal nuclei, mitochondria, and the endoplasmic reticulum (ER). Palade was excluded from ER model training because the ER could not be reliably identified.

Training was performed using the proprietary AI toolkit implemented in ZEISS Arivis Pro (v4.5.0) using the same software configuration and training workflow described previously [26]. The software implements a U-Net-based architecture; however, detailed architectural parameters and internal optimization settings, including optimizer, learning rate, loss function, batch size, and internal input-tile dimensions, are not exposed to the user. The training schedule is internally defined by the software and was not user-adjustable. To artificially expand the morphological variance of the training dataset and prevent overfitting, standard geometric data augmentations—including random rotations, reflections, and scaling—were applied natively to the sub-tiles during the Arivis Pro deep learning training phase. Computational pre-processing, model training, and subsequent inference were performed on a dedicated workstation provided by Carl Zeiss S.p.A. (Milan, Italy), equipped with an NVIDIA Quadro RTX 4000 GPU (8 GB VRAM), 64 GB of RAM, and an Intel Xeon CPU.

Separate models were generally trained for individual structures. Nuclei and satellite glial cells were segmented using a single jointly trained model, whereas mitochondria and ER were segmented using separate models. Model development typically used up to approximately 20 representative image planes sampled across the staining preparations included for the corresponding target structure.

All training and evaluation annotations were generated manually by the same operator. Ground-truth masks used for quantitative evaluation were drawn before inspection of the corresponding model predictions, thereby preventing prediction-guided modification of the evaluation masks. Because annotations were produced by a single annotator, inter-annotator agreement was not assessed.

The performance of the automated segmentation models was quantitatively evaluated using two rigorous spatial overlap metrics: the Intersection over Union (IoU), also known as the Jaccard Index, and the Dice similarity coefficient (DSC). Both metrics quantify the topological accuracy of the predicted segmentation masks against the manually annotated ground truth by calculating true positives (TP), false positives (FP), and false negatives (FN) at the pixel level.

The intersection over union calculates the ratio of the area of overlap between the predicted and ground truth masks to the area of their union, heavily penalizing both under- and over-segmentation. It is mathematically defined as

(2) $$\mathrm{IoU}=\frac{TP}{TP+FP+FN}$$

The Dice similarity coefficient is defined as the harmonic mean of precision and recall. It generally yields a slightly higher numerical value than the IoU and is particularly effective for evaluating complex geometries, such as the thin, branching networks of the ER and the multi-layered SGC sheath. It is mathematically expressed as

(3) $$\mathrm{DSC}\;=\;\frac{2\times TP}{2\times TP+FP+FN}$$

To evaluate segmentation performance across different Z-positions within each cellular volume, quantitative evaluation was performed using a Z-axis-stratified sampling strategy. For each of the three protocols, a representative subset of 30 images was extracted from the fully cropped DRG stacks (10 images sampled from the top, 10 from the middle, and 10 from the bottom of the cellular volume). Manual ground truth (GT) annotations, overall metric calculations, and the generation of visual evaluation maps were exclusively performed on these 30-image subsets. The 30 evaluation sections selected for each staining protocol were excluded from model training and all subsequent active-learning iterations. Training images were selected independently from the remaining sections. Because training and evaluation originated from the same volumetric datasets, spatially adjacent sections were not systematically excluded. The reported metrics should therefore be interpreted as within-volume segmentation validation rather than fully independent cross-volume generalization.

To visually interpret these pixel-level metrics and isolate regional segmentation discrepancies, a custom macro in Fiji (ImageJ v.1.54f) was utilized. This macro systematically processed the exported binary ground truth and model prediction masks from the 30-image subsets to calculate and generate the spatial accuracy overlays (visualizing true positives, false positives, and false negatives), as well as the targeted spatial difference maps (subtraction images) presented in the qualitative results.

DSC and IoU were calculated independently for each sampled section and are reported as mean ± SD across the 30 sections. Individual serial sections represent repeated observations within the same volumetric dataset and were not treated as independent biological replicates. Because adjacent sections within an SBF-SEM stack are spatially correlated, these values are presented descriptively and no inferential statistical comparison between staining protocols was performed.

The trained models were subsequently applied throughout the complete cropped SBF-SEM stacks to generate the volumetric reconstructions shown in Figure 1. Quantitative model validation, however, was performed at the two-dimensional section level using the manually annotated Z-axis-stratified evaluation subsets.

## 3. Results

### 3.1. 3D Volumetric Reconstructions of DRG Sensory Neuron Units

To compare the suitability of the three staining protocols for volumetric imaging, SBF-SEM datasets were first assessed for image contrast and subsequently processed using a standardized deep-learning segmentation pipeline. This workflow was used to reconstruct individual DRG sensory neuron units and visualize the spatial relationships between the neuronal soma, intracellular organelles, and surrounding SGCs.

A detailed visual comparison combining raw 2D orthoslices with their corresponding 3D volumetric models (Figure 1) demonstrates differences in image contrast and the resulting morphological information between the three staining methods. In the Palade protocol dataset (Figure 1A), the raw overview and magnified region of interest (Figure 1(A1)) provide sufficient baseline contrast to identify the nucleus (N), mitochondria (m), and the encapsulating SGC layer. However, the ER could not be reliably distinguished in these images because of insufficient membrane contrast. Consequently, the resulting 3D reconstruction (Figure 1(A2)) successfully renders the nucleus, mitochondria, and SGC sheath but lacks any volumetric representation of the ER network.

The Ellisman protocol (Figure 1B) yielded high-contrast rOTO membrane staining. The magnified orthoslice (Figure 1(B1)) clearly resolves the fragmented tubule network of the ER alongside the nucleus, mitochondria, and SGC boundaries. The corresponding 3D reconstruction (Figure 1(B2)) included all targeted structures, including the densely interconnected ER network.

Similarly, the Uranyl-free (X-solution) protocol (Figure 1C) showed clear membrane and chromatin contrast without uranyl-containing reagents. The detailed raw ultrastructure (Figure 1(C1)) clearly delineates the ER, with ER structures readily distinguishable in the raw images. The resulting 3D reconstruction (Figure 1(C2)) included the nucleus, mitochondria, SGC sheath, and ER network, indicating that the image contrast was sufficient for segmentation of these structures in 3D.

### 3.2. Quantitative Segmentation Performance Across DRG Domains

To quantify the segmentation performance of each staining method, identical deep-learning models were applied to all three DRG datasets. Prior to model application, all image stacks were processed using the fixed preprocessing workflow described in Section 2.2. The models were applied to four target structures: satellite glial cells (SGCs), nuclei, mitochondria, and the endoplasmic reticulum (ER).

Model performance was evaluated using two standard overlap metrics: intersection over union (IoU) and Dice similarity coefficient (DSC) (Table 1). Both metrics quantify the overlap between the automated predictions and manually annotated ground-truth masks, with values ranging from 0 (no overlap) to 1 (complete overlap).

The resulting quantitative data reveal differences in segmentation performance among the three staining protocols. While nuclear segmentation remained highly robust across all protocols, the X-solution produced the highest DSC and IoU values for SGCs and mitochondria. The ER could not be quantified in the Palade dataset because of insufficient image contrast.

### 3.3. Automated Segmentation of Satellite Glial Cells (SGCs)

Segmentation of the multilayered SGC sheath was challenging because of its complex geometry and close apposition to surrounding structures. Quantitative evaluation (Table 1) revealed that the uranyl-free (X-solution) protocol showed the highest overlap with the ground-truth masks, ranking first with a Dice similarity coefficient (DSC) of 0.9072 ± 0.0514 and an IoU of 0.8334 ± 0.0882. The standard Palade protocol ranked second (DSC: 0.8881 ± 0.0247; IoU: 0.7994 ± 0.0398), while the heavy-metal Ellisman protocol performed the poorest, ranking last across both metrics (DSC: 0.7921 ± 0.0412; IoU: 0.6575 ± 0.0556).

To visually interpret these performance differences, subtraction error maps (Prediction—GT) were generated to visualize false-positive predictions (false positives). As demonstrated in Figure 2, the Palade protocol (Row A) exhibited the most conservative boundary predictions, resulting in the least false-positive leakage (Figure 2(A3)). In contrast, both the Ellisman (Figure 2(B3)) and uranyl-free (Figure 2(C3)) stains showed occasional boundary over-segmentation, where the model slightly expanded the SGC mask into adjacent high-contrast regions. However, despite displaying some false-positive leakage comparable to the Ellisman dataset, the uranyl-free protocol achieved the highest overall metrics, suggesting that the higher overlap scores were primarily associated with more complete coverage of the SGC regions, despite the presence of some false-positive predictions.

### 3.4. Automated Segmentation of Neuronal Nuclei

In contrast to the SGCs, nuclear segmentation was highly successful across all three en bloc staining protocols. All three methods achieved DSC values above 0.98 (Table 1). The Ellisman protocol produced the highest DSC (0.9921 ± 0.0060) and IoU (0.9843 ± 0.0117) values, closely followed by the Palade (DSC: 0.9826 ± 0.0090; IoU: 0.9659 ± 0.0172) and Uranyl-free (DSC: 0.9812 ± 0.0094; IoU: 0.9632 ± 0.0180) preparations.

The strong and relatively consistent chromatin contrast across all three protocols likely facilitated nuclear segmentation, resulting in high overlap scores. However, a close examination of the prediction–ground-truth difference maps reveals a subtle distinction in boundary precision. While the heavy-metal Ellisman (Figure 3(B3)) and Uranyl-free (Figure 3(C3)) preparations showed few false-positive regions and close agreement between the predicted and ground-truth boundaries, the standard Palade protocol (Figure 3(A3)) exhibited a distinct ring of false-positive predictions around the nuclear boundary. This pattern suggests a slight overestimation of the nuclear boundary in the Palade dataset, potentially reflecting lower contrast between the nucleus and surrounding structures compared with the rOTO and X-solution preparations.

### 3.5. Automated Segmentation of Mitochondria

Unlike nuclear segmentation, mitochondrial segmentation showed substantial differences among the three staining protocols. While the Ellisman protocol produced high membrane contrast, quantitative evaluation showed higher segmentation scores for the Uranyl-free protocol, which achieved a higher Dice score of 0.9242 ± 0.0079 and an IoU of 0.8592 ± 0.0136 (Table 1). In comparison, Ellisman achieved a DSC of 0.8123 ± 0.0272 with an IoU of 0.6847 ± 0.0380, while Palade achieved a DSC of 0.7093 ± 0.0375 with IoU of 0.5508 ± 0.0452.

A detailed examination of the spatial difference maps (Prediction minus Ground Truth) revealed differences in the distribution of false-positive and false-negative regions. The Uranyl-free protocol (Figure 4(C3)) and the Ellisman protocol (Figure 4(B3)) showed occasional localized false-positive regions of similar appearance. The higher scores for the X-solution were associated with more complete overlap with the ground-truth mitochondrial masks and fewer false-negative regions. The visually more homogeneous appearance of the X-solution images may have contributed to this difference, although contrast uniformity was not quantitatively evaluated. The Palade protocol (Figure 4(A3)) showed fewer false-positive regions, but also substantially lower mitochondrial segmentation scores, consistent with weaker mitochondrial contrast and reduced representation of the mitochondrial network.

### 3.6. Automated Segmentation of the Endoplasmic Reticulum (ER)

The ER was the most challenging structure to segment because of its sparse, fragmented, and tubular morphology. As previously established in the 3D reconstructions, the ER could not be reliably distinguished in the Palade dataset due to a lack of deep membrane contrast. Therefore, the Palade dataset was excluded from quantitative ER segmentation analysis.

Among the two protocols that produced sufficient ER contrast, the Uranyl-free method produced higher DSC and IoU values than the Ellisman protocol (DSC: 0.5941 ± 0.0433 vs. 0.4989 ± 0.0543; IoU: 0.4238 ± 0.0436 vs. 0.3340 ± 0.0471). The morphological complexity underlying these lower overall scores is evident in the manual ground truth annotations (Figure 5(A1,B1)), which detail a highly convoluted and discontinuous network.

When visually evaluating the spatial accuracy maps, a difference in the balance between false-positive and false-negative predictions becomes apparent. The Ellisman preparation (Figure 5(A2)) showed substantial under-segmentation; the overlay is dominated by false negatives (red), indicating the model failed to detect the vast majority of the delicate ER network. The sparse subtraction map (Figure 5(A3)) reflects the low number of false-positive predictions, which is consistent with the conservative segmentation and extensive under-prediction of the ER.

In contrast, the Uranyl-free model showed greater coverage of the ER network. The spatial accuracy map (Figure 5(B2)) exhibits a higher proportion of true-positive regions (yellow), capturing the continuity of the ER tubules much more effectively. However, the broader predicted regions also resulted in more false-positive predictions in the subtraction map (Figure 5(B3)). Ultimately, despite generating more false positives, the Uranyl-free method’s ability to successfully delineate the actual ER network results in fewer false-negative regions, which is consistent with the higher DSC and IoU values.

## 4. Discussion

The combination of volumetric electron microscopy and deep learning provides an approach for reconstructing complex neural tissues in three dimensions. However, segmentation performance depends strongly on the contrast and image quality produced by the underlying sample preparation. In this study, we systematically evaluated three en bloc staining protocols—Palade, Ellisman, and uranyl-free (X-solution)—to compare their suitability for automated segmentation and 3D reconstruction of dorsal root ganglia (DRG) sensory neurons under a common preprocessing and deep-learning workflow. Our findings demonstrate that the staining conditions that produce the strongest visual contrast do not necessarily provide the best conditions for automated segmentation.

### 4.1. Visual Contrast Does Not Predict Segmentation Performance

Traditional electron microscopy staining protocols have generally been optimized to provide strong contrast between cellular membranes and surrounding structures [26,27,28,29]. The Ellisman (rOTO) protocol illustrates this approach, producing high-contrast images with strong membrane definition. However, in the present datasets, the strong membrane contrast of the Ellisman preparation did not translate into the highest segmentation performance. Mitochondrial and satellite glial cell (SGC) segmentation yielded lower DSC and IoU values for the Ellisman preparation than for the X-solution dataset [26,30,31].

The Ellisman dataset exhibited visually apparent regions of very high local contrast and occasional precipitate-like features [31]. Such image heterogeneity could potentially contribute to segmentation errors by introducing features resembling biological boundaries, thereby increasing false-positive or false-negative predictions [32,33]. However, the contribution of these image characteristics to model performance was not quantitatively assessed in the present study.

In contrast, the uranyl-free (X-solution) images appeared visually more homogeneous and achieved higher DSC and IoU values for several of the more complex structures examined in this study. One possible explanation is that more consistent image features within and around the target structures facilitated their separation from the surrounding tissue by the segmentation model. For example, the X-solution produced substantially higher mitochondrial and SGC segmentation scores than the Ellisman protocol, despite the presence of some false-positive regions. Thus, the present results indicate that the strongest visual contrast does not necessarily correspond to the highest segmentation overlap and suggest that other image characteristics, including local contrast consistency and structural specificity, may also influence model performance [26,34].

It should also be noted that all three datasets were processed using the same fixed preprocessing workflow before model inference, including non-local means denoising, Z-score normalization, CLAHE, and sharpening. The contribution of the individual preprocessing steps and their potential interaction with staining chemistry were not independently evaluated in the present study. Accordingly, the differences reported here should be interpreted as the performance of the three staining preparations within the common preprocessing and segmentation workflow rather than as isolated effects of staining chemistry alone. The rationale and previous evaluation of this preprocessing strategy have been described previously [26].

### 4.2. Structure-Specific Segmentation Performance

The differences in segmentation performance among the four target structures indicate that the effect of staining chemistry depends on the morphology and contrast characteristics of the structure being segmented:

- **Nuclei:** Nuclear segmentation was the most consistent across the three staining protocols. The strong chromatin contrast provided a readily distinguishable feature for nuclear segmentation across all three protocols, yielding Dice scores exceeding 0.98. Although the Palade protocol showed a slight tendency toward false-positive predictions around the nuclear boundary, the overall segmentation performance remained high across all three protocols. These results indicate that nuclear segmentation was relatively insensitive to the choice of staining protocol.
- **Satellite glial cells (SGCs):** The multilayered SGC sheath has a complex geometry and close association with adjacent structures, making accurate boundary segmentation more challenging. The Palade protocol generated fewer false-positive regions but also showed more conservative segmentation, resulting in less complete representation of the manually annotated SGC regions. The X-solution produced the highest DSC and IoU values for SGCs. Its images also appeared visually more homogeneous than the Ellisman dataset; however, because contrast uniformity was not quantitatively evaluated, the relationship between this image characteristic and the higher segmentation scores remains speculative.
- **Mitochondria:** Differences between the staining protocols were also pronounced for mitochondrial segmentation. The X-solution produced the highest DSC and IoU values despite the strong membrane contrast observed in the Ellisman dataset. The visually more homogeneous appearance of the X-solution images may have contributed to discrimination of mitochondrial regions from the surrounding cytoplasm, although this was not quantitatively tested. In contrast, the Ellisman dataset showed greater false-positive and false-negative segmentation, and the Ellisman images also exhibited greater local contrast heterogeneity and precipitate-like features. Whether these characteristics directly contributed to the segmentation errors cannot be determined from the present analysis.

### 4.3. Challenges in ER Segmentation

The ER represented the most challenging target for automated segmentation because of its sparse, tubular, and highly fragmented morphology [35,36]. The Palade dataset could not be used for quantitative ER segmentation because the ER was not reliably distinguishable in the raw images, indicating that the membrane contrast provided by this protocol was insufficient for reconstruction of the ER network [37].

Among the remaining protocols, the comparison revealed a difference in the balance between false-positive and false-negative predictions. The Ellisman model showed substantial under-segmentation, with a high proportion of false-negative regions. Although this resulted in relatively few false-positive regions in the subtraction maps, the predicted masks captured only a limited portion of the ER network. In contrast, the Uranyl-free model detected a substantially larger proportion of the ER structures. This increased coverage was accompanied by more false-positive regions, but the resulting DSC and IoU values were higher than those obtained with the Ellisman protocol. Together, these findings indicate that, within the datasets and workflow examined here, the X-solution provided more complete automated segmentation of the sparse and morphologically complex ER network than the Ellisman preparation [35,37].

### 4.4. Study Limitations

Several limitations should be considered when interpreting the present findings. First, the staining protocols were represented by a limited number of biological specimens and volumetric datasets. Therefore, the observed differences should primarily be interpreted as technical performance differences within the datasets examined rather than as population-level estimates of staining performance. Validation across additional animals, DRGs, tissue blocks, and independent staining batches will be required to establish the reproducibility and generalizability of the protocol ranking.

Second, although the 30 sections used for the quantitative evaluation were excluded from model training and subsequent active-learning iterations, the training and evaluation images originated from the same volumetric datasets, and spatially adjacent sections were not systematically excluded from training. The reported DSC and IoU values therefore represent within-volume validation rather than truly independent cross-volume generalization.

Third, quantitative validation was performed on Z-axis-stratified two-dimensional sections rather than fully annotated three-dimensional ground-truth volumes. The trained models were applied throughout the complete image stacks to generate the 3D reconstructions, but the reported overlap metrics specifically quantify section-level segmentation agreement. Future studies incorporating independently annotated volumetric ground truth and 3D overlap or boundary-based metrics would provide a more direct assessment of volumetric segmentation accuracy.

Finally, all datasets underwent the same preprocessing workflow before inference. Because the individual preprocessing operations were not independently evaluated in the present study, an interaction between staining chemistry and preprocessing cannot be excluded.

## 5. Conclusions

This study demonstrates that staining protocols optimized for conventional electron microscopy do not necessarily provide equivalent performance for automated segmentation and 3D reconstruction. Comparison of the Palade, Ellisman, and uranyl-free X-solution preparations revealed clear structure-dependent differences in segmentation performance. Nuclear segmentation remained highly accurate across all three protocols, whereas larger differences were observed for satellite glial cells (SGCs), mitochondria, and the endoplasmic reticulum (ER).

In the datasets examined, the Palade preparation provided sufficient contrast for reliable nuclear and mitochondrial segmentation but did not provide adequate membrane contrast for reliable identification and quantitative segmentation of the ER. The Ellisman preparation produced strong ultrastructural contrast and enabled segmentation of all evaluated structures, although the corresponding images exhibited local intensity heterogeneity and precipitate-like features, together with increased false-positive and false-negative regions for several complex structures. The X-solution produced the highest DSC and IoU values for SGCs and mitochondria and higher ER segmentation scores than the Ellisman preparation, while maintaining high nuclear segmentation accuracy.

Overall, these findings indicate that the strongest visual ultrastructural contrast does not necessarily correspond to the highest segmentation overlap under a common preprocessing and deep-learning workflow. The X-solution therefore represents a promising uranyl-free alternative for AI-assisted analysis and volumetric reconstruction of DRG tissue, particularly when the structures of interest have complex or fragmented morphology. Its avoidance of uranyl acetate also provides a practical advantage for laboratories seeking alternative staining reagents. However, the present results should be interpreted within the biological specimens, imaging datasets, and computational workflow examined here. Further validation across additional specimens, staining batches, imaging conditions, and independently annotated volumetric datasets will be required to establish the reproducibility and generalizability of these findings.

## Figures and Tables

**Figure 1:**
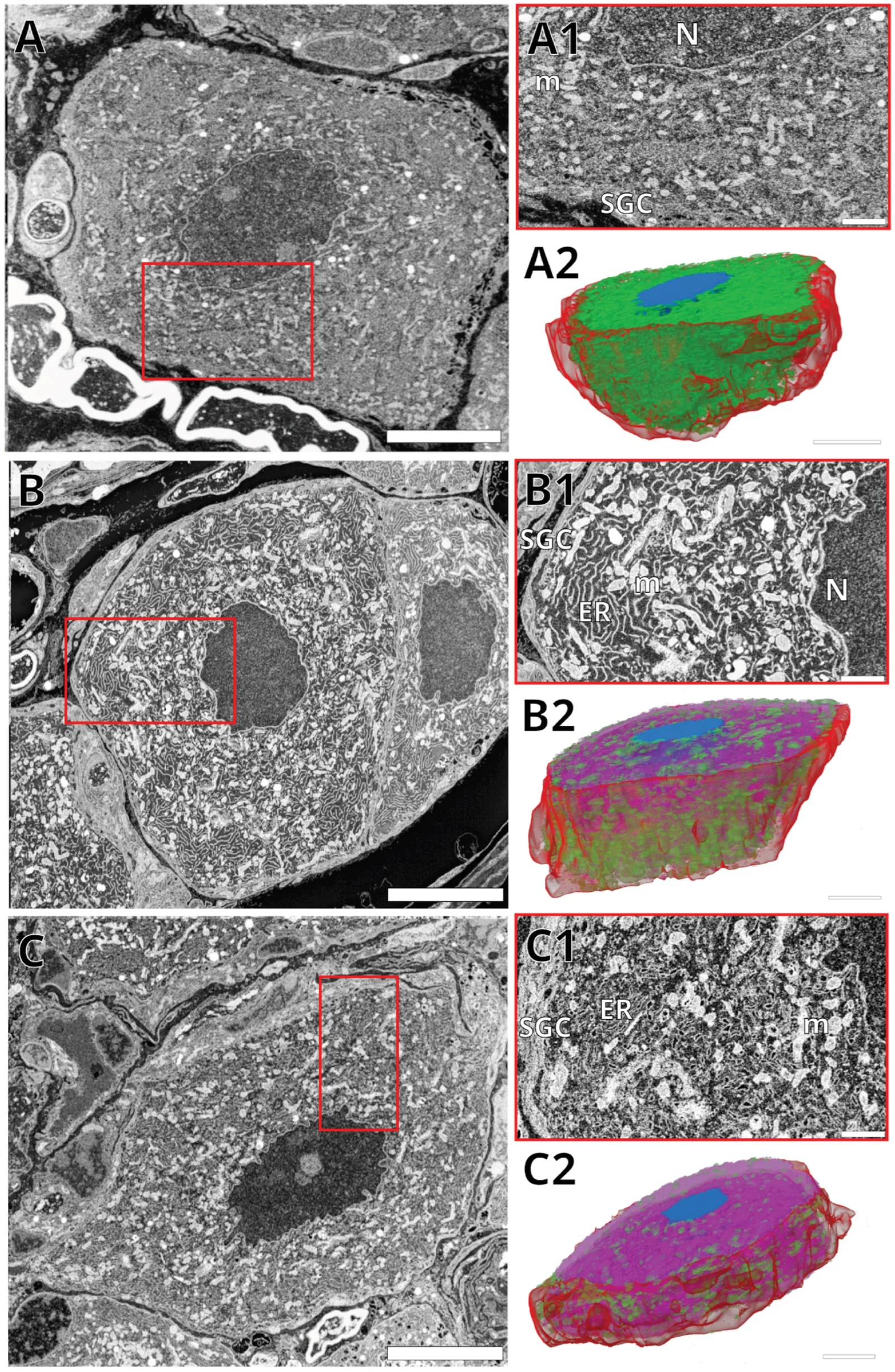
Correlative 2D Ultrastructure and 3D Volumetric Reconstructions of DRG Sensory Neuron Units. Comparative evaluation of individual DRG cells processed with identical deep-learning segmentation pipelines across the three evaluated en bloc staining protocols. (**A**) Palade Protocol: Raw SBF-SEM orthoslice (**A**) and magnified Region of Interest (**A1**) showing the neuronal nucleus (N), mitochondria (m), and satellite glial cell (SGC). The 3D reconstruction (**A2**) displays the nucleus (blue), mitochondrial network (green), and the enveloping SGC sheath (red). The Endoplasmic Reticulum (ER) was not included in the reconstruction because it could not be reliably distinguished in the Palade images. (**B**) Ellisman Protocol: Raw orthoslice (**B**) and magnified ROI (**B1**) demonstrating high-contrast rOTO membrane staining. The corresponding 3D reconstruction (**B2**) displays the complete morphological suite: nucleus (blue), mitochondria (green), SGC sheath (red), and the highly resolved, tubulated network of the ER (magenta). (**C**) Uranyl-free (X-solution) Protocol: Raw orthoslice (**C**) and magnified ROI (**C1**) demonstrating excellent membrane and chromatin contrast without radioactive reagents. The 3D reconstruction (**C2**) successfully captures the complete ultrastructure: nucleus (blue), mitochondria (green), SGC (red), and ER network (magenta), matching the volumetric preservation of the heavy-metal standard. Rendering parameters: Volumetric models were rendered in native (highest) quality. Transparency settings were optimized for deep-tissue visualization: mitochondria (27% opacity), ER (10% opacity), and SGCs rendered as a glass material (100% opacity). Scale bars: 10 µm in main panels (**A**–**C**) and 3D renders (**A2**–**C2**); 2 µm in magnified panels (**A1**–**C1**).

**Figure 2:**
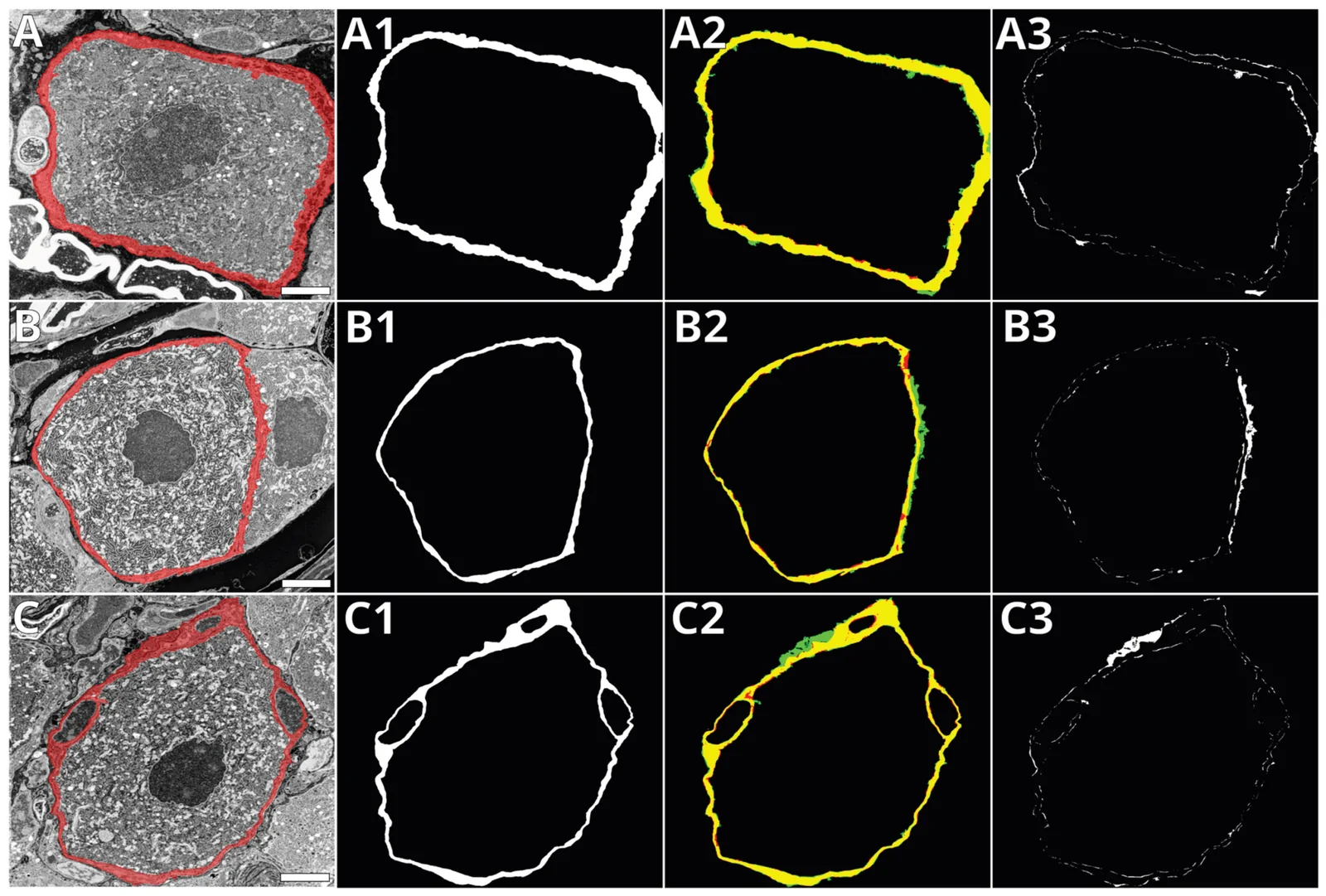
Spatial accuracy evaluation of Satellite Glial Cell (SGC) segmentation. Representative regions of SGC sheath segmentation evaluated against manually annotated ground truth across three diverse en bloc staining preparations: (Row A) Palade, (Row B) Ellisman, and (Row C) Uranyl-free (X-solution). (**A**–**C**) Standardized SBF-SEM orthoslices displaying the model’s spatial predictions overlaid in red. (**A1**–**C1**) Corresponding manually annotated binary ground truth masks. (**A2**–**C2**) Spatial accuracy maps generated by directly overlaying the ground truth (red) and model prediction (green). In these composites, yellow represents True Positives (spatial consensus), red indicates False Negatives (ground truth structures missed by the model), and green indicates False Positives (model over-segmentation). (**A3**–**C3**) Spatial difference maps generated by subtracting the ground truth from the AI prediction (Prediction—GT), highlighting the precise boundaries of false-positive segmentation discrepancies. Scale bars: 5 µm.

**Figure 3:**
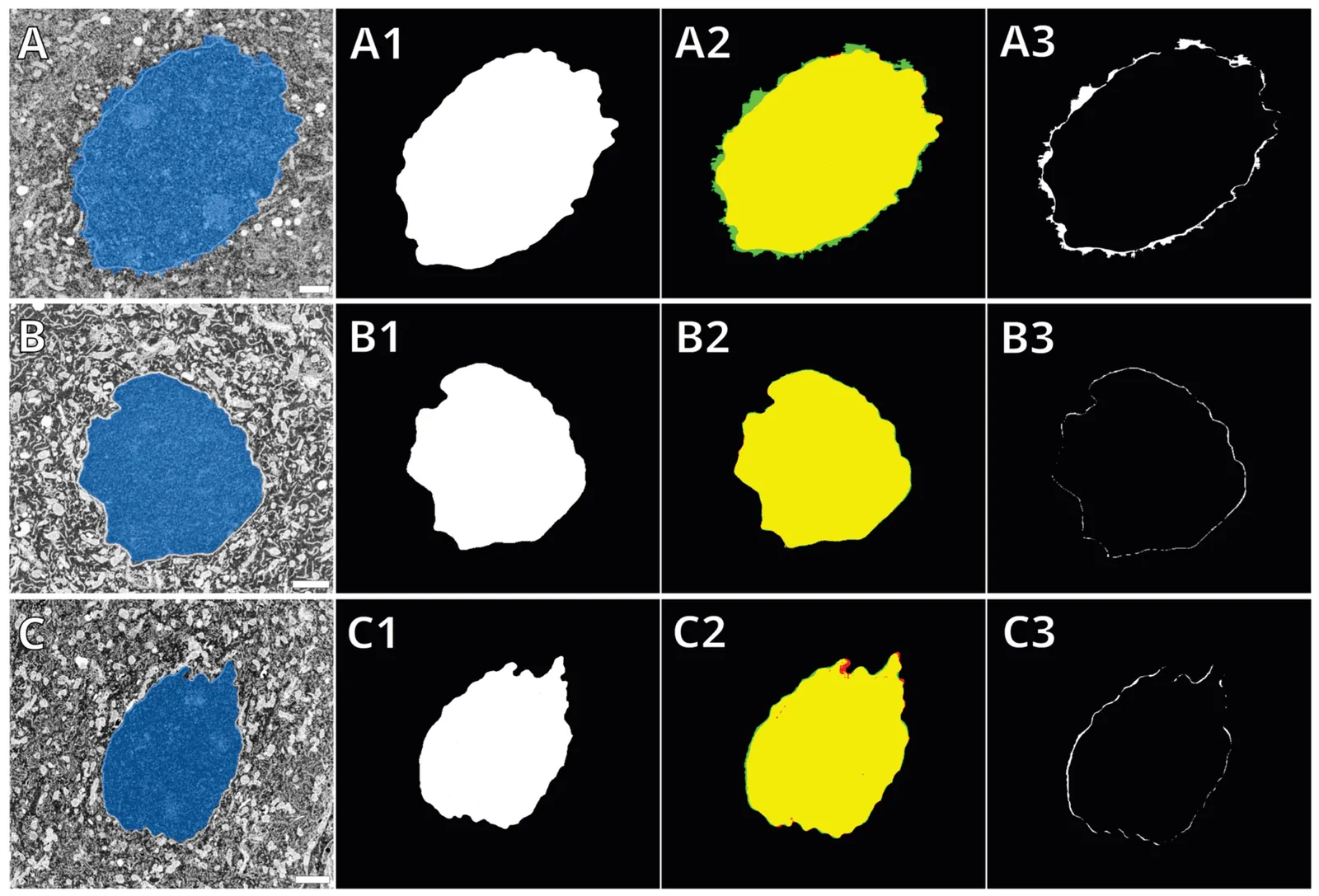
Spatial accuracy evaluation of Neuronal Nuclei segmentation. Representative regions of neuronal nucleus segmentation evaluated against manually annotated ground truth across three diverse en bloc staining preparations: (Row A) Palade, (Row B) Ellisman, and (Row C) Uranyl-free (X-solution). (**A**–**C**) Standardized SBF-SEM orthoslices displaying the model’s spatial predictions overlaid in blue. (**A1**–**C1**) Corresponding manually annotated binary ground truth masks. (**A2**–**C2**) Spatial accuracy maps generated by directly overlaying the ground truth (red) and model prediction (green). In these composites, yellow represents True Positives (spatial consensus), red indicates False Negatives (ground truth structures missed by the model), and green indicates False Positives (model over-segmentation). (**A3**–**C3**) Spatial difference maps generated by subtracting the ground truth from the AI prediction (Prediction—GT), highlighting the precise boundaries of false-positive segmentation discrepancies. Scale bars: 2 µm.

**Figure 4:**
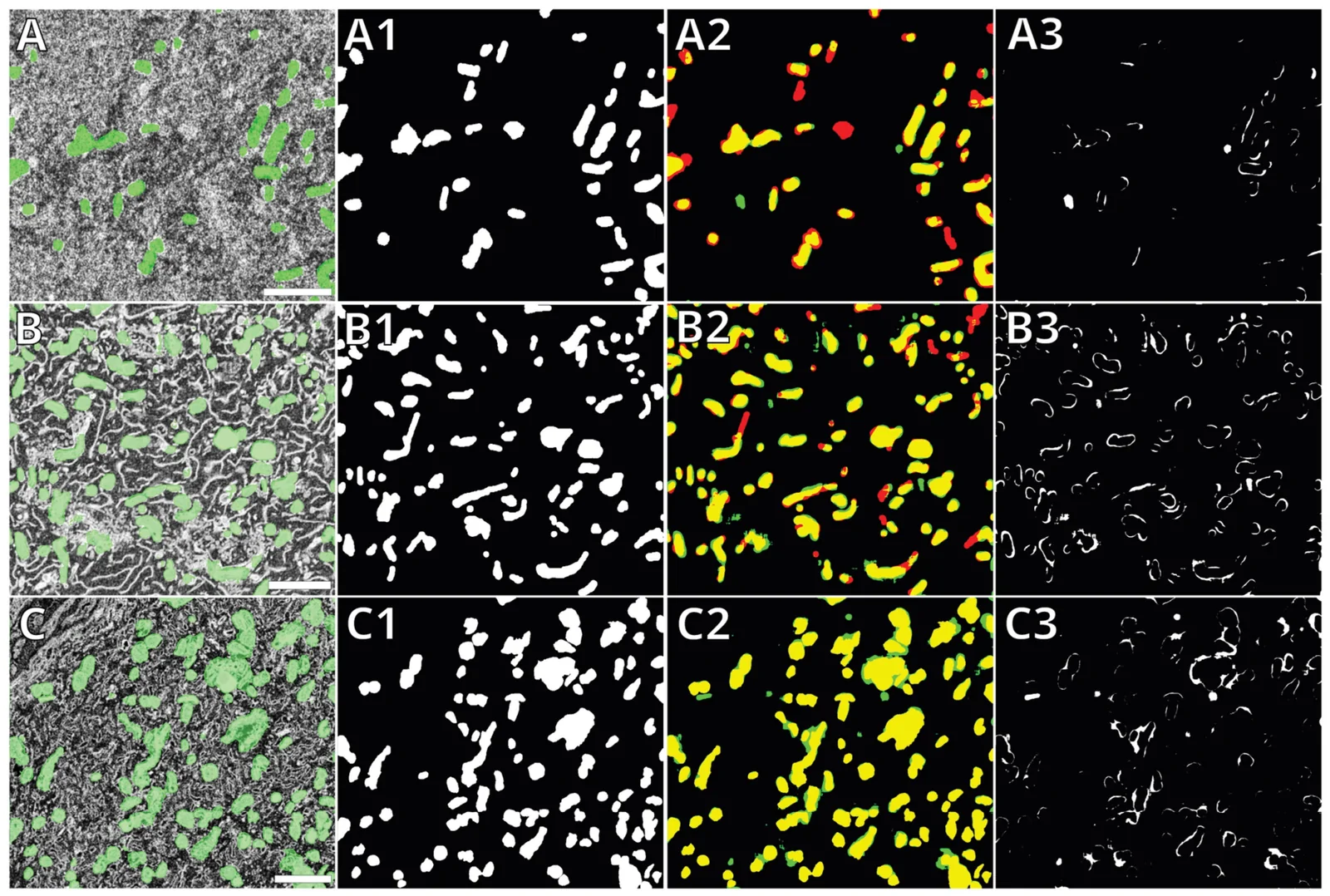
Spatial accuracy evaluation of Mitochondrial network segmentation. Representative regions of DRG mitochondria segmentation evaluated against manually annotated ground truth across three diverse en bloc staining preparations: (Row A) Palade, (Row B) Ellisman, and (Row C) Uranyl-free (X-solution). (**A**–**C**) Standardized SBF-SEM orthoslices displaying the model’s spatial predictions overlaid in green. (**A1**–**C1**) Corresponding manually annotated binary ground truth masks. (**A2**–**C2**) Spatial accuracy maps generated by directly overlaying the ground truth (red) and model prediction (green). In these composites, yellow represents True Positives (spatial consensus), red indicates False Negatives (ground truth structures missed by the model), and green indicates False Positives (model over-segmentation). (**A3**–**C3**) Spatial difference maps generated by subtracting the ground truth from the AI prediction (Prediction—GT). Note that while the Ellisman (**B3**) and Uranyl-free (**C3**) preparations exhibit comparable levels of localized false-positive dispersion, the Uranyl-free approach achieves higher overall overlap, whereas the Palade protocol (**A3**) shows fewer false positives but lower overall agreement with the manually annotated mitochondrial masks. Scale bars: 2 µm.

**Figure 5:**
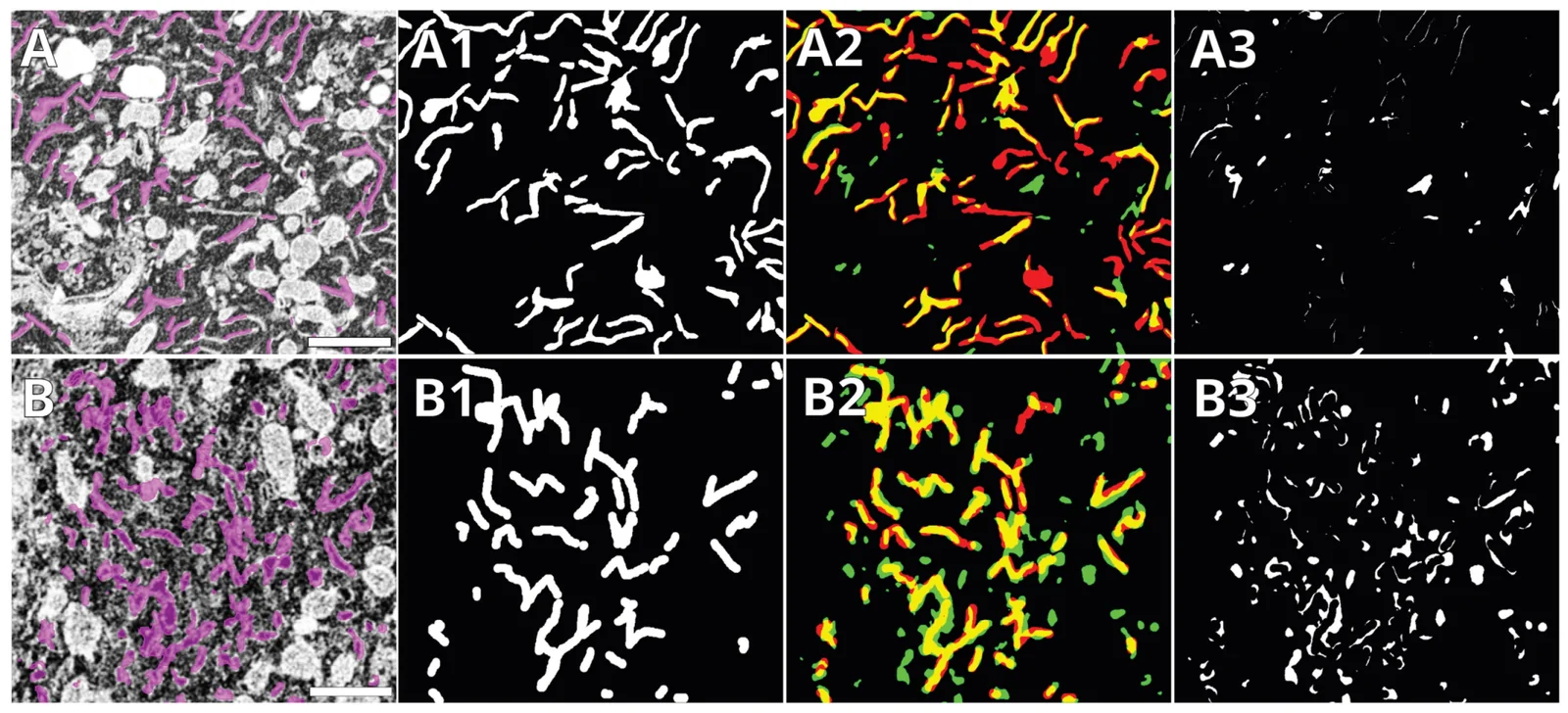
Spatial accuracy evaluation of Endoplasmic Reticulum (ER) segmentation. Representative regions of ER tubule segmentation evaluated against manually annotated ground truth across the highly conductive en bloc staining preparations: (Row A) Ellisman and (Row B) Uranyl-free (X-solution). Note: The Palade protocol is omitted due to indiscernible ER ultrastructure. (**A**,**B**) Standardized SBF-SEM orthoslices displaying the model’s spatial predictions overlaid in magenta. (**A1**,**B1**) Corresponding manually annotated binary ground truth masks. (**A2**,**B2**) Spatial accuracy maps generated by directly overlaying the ground truth (red) and model prediction (green). In these composites, yellow represents True Positives (spatial consensus), red indicates False Negatives (ground truth structures missed by the model), and green indicates False Positives (model over-segmentation). (**A3**,**B3**) Spatial difference maps generated by subtracting the ground truth from the AI prediction (Prediction—GT). The visual analysis reveals severe under-segmentation in the Ellisman sample, leading to extensive false negative regions (**A2**) and a sparse false-positive map (**A3**). Conversely, the X-solution model demonstrates higher sensitivity, yielding more true positives (**B2**) at the cost of a denser false-positive distribution (**B3**). Scale bars: 1 µm.

**Table 1:** Quantitative segmentation metrics by DRG domain. Values represent descriptive mean ± SD calculated across 30 Z-axis-stratified evaluation sections for each staining dataset. Sections represent repeated observations within the corresponding volumetric dataset rather than independent biological replicates.

DRG Domain	Structure	IoU (Mean ± SD)	DSC (Mean ± SD)
Ellisman	Endoplasmic Reticulum	0.3340 ± 0.0471	0.4989 ± 0.0543
	Mitochondria	0.6847 ± 0.0380	0.8123 ± 0.0272
	Nucleus	0.9843 ± 0.0117	0.9921 ± 0.0060
	Satellite cells	0.6575 ± 0.0556	0.7921 ± 0.0412
Palade	Endoplasmic Reticulum	N/A	N/A
	Mitochondria	0.5508 ± 0.0452	0.7093 ± 0.0375
	Nucleus	0.9659 ± 0.0172	0.9826 ± 0.0090
	Satellite cells	0.7994 ± 0.0398	0.8881 ± 0.0247
Uranyl-free	Endoplasmic Reticulum	0.4238 ± 0.0436	0.5941 ± 0.0433
	Mitochondria	0.8592 ± 0.0136	0.9242 ± 0.0079
	Nucleus	0.9632 ± 0.0180	0.9812 ± 0.0094
	Satellite cells	0.8334 ± 0.0882	0.9072 ± 0.0514

## Data Availability

The custom Python-based pre-processing script developed for this study and the models are publicly available on GitHub at https://github.com/Nochebald/EM-raw-image-standardization.git (accessed on 28 August 2026). The raw electron microscopy datasets are available from the corresponding author upon reasonable request.
